# Computation of robustly stabilizing PID controllers for interval systems

**DOI:** 10.1186/s40064-016-2341-z

**Published:** 2016-05-20

**Authors:** Radek Matušů, Roman Prokop

**Affiliations:** Centre for Security, Information and Advanced Technologies (CEBIA – Tech), Faculty of Applied Informatics, Tomas Bata University in Zlín, nám. T. G. Masaryka 5555, 760 01 Zlín, Czech Republic

**Keywords:** Robust stabilization, PID control, PI control, Interval systems, Oblique wing aircraft

## Abstract

The paper is focused on the computation of all possible robustly stabilizing Proportional-Integral-Derivative (PID) controllers for plants with interval uncertainty. The main idea of the proposed method is based on Tan’s (et al.) technique for calculation of (nominally) stabilizing PI and PID controllers or robustly stabilizing PI controllers by means of plotting the stability boundary locus in either P-I plane or P-I-D space. Refinement of the existing method by consideration of 16 segment plants instead of 16 Kharitonov plants provides an elegant and efficient tool for finding all robustly stabilizing PID controllers for an interval system. The validity and relatively effortless application of presented theoretical concepts are demonstrated through a computation and simulation example in which the uncertain mathematical model of an experimental oblique wing aircraft is robustly stabilized.

## Background

The Proportional-Integral-Derivative (PID) control algorithms and their simplifications (P, I, PD and especially PI) comprise the great majority of contemporary industrial control applications. It has been reported that they represent over 90 % of all practically applied controllers in process control (Åström and Hägglund 1995; O‘Dwyer 2003). Thus, despite the existence of many more sophisticated control design methods and modern approaches (see e.g. (Selma and Chouraqui 2013) for the example of neuro-fuzzy control, (Ahmed et al. 2014) for the static synchronous series compensator based damping control, (Ibraheem et al. 2014) for automatic generation control, or (Shang 2016) for the stochastic consensus problems for multi-agent systems over Markovian switching networks with time-varying delays and topology uncertainties), the effective tuning of PI and PID controllers is still very topical because it can bring significant saving on energy as well as expenses. Evidently, the systematic research on the application of the PI(D) controllers under various conditions of uncertainty contributes to this mosaic.

Obviously, the stability is the first and most critical requirement of all control applications. However, the real-life control circumstances differ from the ideal nominal ones and so the uncertainty of the mathematical models has to be frequently taken into considerations. The attention of many researchers has been focused on the investigation of robust stability for systems with parametric uncertainty—see e.g. (Barmish 1994; Bhattacharyya et al. 1995, 2009; Matušů and Prokop 2011). Typical problem of practical PI(D) controller design is to ensure, that the calculated controller will guarantee stability not only for one assumed nominal controlled system but also for the whole family of systems described by a model with parametric uncertainty. Such closed-loop control system is called as “robustly stable” and the controller itself is then robustly stabilizing one.

An array of techniques for calculation of (nominally) stabilizing PI and PID controllers have been already published, such as rules presented in (Söylemez et al. 2003), the Tan’s method described in (Tan and Kaya 2003; Tan et al. 2006) or the Kronecker summation method from (Fang et al. 2009). Furthermore, these methods have been also extended for robust stabilization of interval plants by their combination with the sixteen plant theorem (Barmish et al. 1992; Barmish 1994). Nevertheless, this extension works only for PI but not for PID controllers.

The main aim of this paper is to present a method for computation of all possible robustly stabilizing PID controllers for interval plants and to demonstrate its serviceability by robust stabilization of an oblique wing aircraft model. More specifically, the goal is to refine the elegant and effective Tan’s method (Tan and Kaya 2003; Tan et al. 2006) by the ideas from (Ho et al. 1998, 2001), i.e. to use 16 segment plants instead of 16 Kharitonov plants, and to make the existing method applicable for computation of robustly stabilizing PID controllers. Previously, the computation of all (nominally) stabilizing PI or PID controllers, robustly stabilizing PI controllers and consequent choice of the specific controller with desired performance on the basis of the desired model method (formerly known as dynamics inversion method) (Vítečková 2000) is shown in (Matušů 2011). Then, the application of Kronecker summation method (Fang et al. 2009) to robust stabilization of a chemical reactor or robust stabilization of a third order nonlinear electronic model is given in (Matušů et al. 2011) or (Matušů et al. 2010a), respectively. The robust stabilization of the same nonlinear electronic plant using the Tan’s method (Tan and Kaya 2003; Tan et al. 2006) is presented e.g. in (Matušů et al. 2010b).

The paper is organized as follows. In “Computation of (nominal) stability regions for PI controllers” section, a graphical method for computation of (nominally) stabilizing PI controllers is recalled. “Computation of (nominal) stability regions for PID controllers” section has the same purpose but for PID controllers. Next, the computation of robustly stabilizing PI controllers for interval plants is presented in “Robust stabilization using PI controllers” section. The key “Robust stabilization using PID controllers” section extends the existing Tan’s (et al.) method, combines it with the segment plants concept and makes it applicable for calculation of robustly stabilizing PID controllers. Further, the extensive “Illustrative example: Robust stabilization of oblique wing aircraft” section confirms the obtained results by means of the simulation example with an experimental oblique wing aircraft model. And finally, “Conclusion” section offers some conclusion remarks.

## Computation of (nominal) stability regions for PI controllers

First, the fundamentals related to computation of (nominal) stability regions for PI controllers are going to be summarized.

**Fig. 1:**
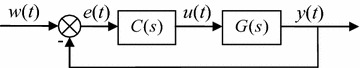
Closed-loop control system

Suppose the classical closed-loop control system according to Fig. 1, where *C*(*s*) represents a controller, *G*(*s*) stands for a controlled system, and signals *w*(*t*), *e*(*t*), *u*(*t*) and *y*(*t*) denote a reference value, tracking (control) error, actuating (control) signal and output (controlled) variable, respectively.

The controller is assumed in the well-known PI form:

1 $$C(s) = k_{P} + \frac{{k_{I} }}{s} = \frac{{k_{P} s + k_{I} }}{s}$$

where *k*_*P*_, *k*_*I*_ represent the proportional and integral gain, respectively. The principal task is to determine the parameters *k*_*P*_, *k*_*I*_ which guarantee stabilization of the controlled plant:

2 $$G(s) = \frac{B(s)}{A(s)}$$

Several effective methods for the computation of stabilizing PI controllers have been already published, e.g. (Söylemez et al. 2003; Tan and Kaya 2003; Tan et al. 2006; Fang et al. 2009). Here, the Tan’s method from (Tan and Kaya 2003; Tan et al. 2006) will be revisited and extended. This graphical approach is based on plotting the stability boundary locus. The substitution of *s* for *jω* in the plant transfer function (2) and subsequent decomposition of the numerator and denominator into their even and odd parts result in:

3 $$G(j\omega ) = \frac{{B_{E} ( - \omega^{2} ) + j\omega B_{O} ( - \omega^{2} )}}{{A_{E} ( - \omega^{2} ) + j\omega A_{O} ( - \omega^{2} )}}$$

Further, expressing the closed-loop characteristic polynomial and equating both real and imaginary parts to zero lead to the relations for the proportional and integral gains *k*_*P*_, *k*_*I*_:

4 $$\begin{aligned} k_{P} (\omega ) & = \frac{{P_{5} (\omega )P_{4} (\omega ) - P_{6} (\omega )P_{2} (\omega )}}{{P_{1} (\omega )P_{4} (\omega ) - P_{2} (\omega )P_{3} (\omega )}} \\ k_{I} (\omega ) & = \frac{{P_{6} (\omega )P_{1} (\omega ) - P_{5} (\omega )P_{3} (\omega )}}{{P_{1} (\omega )P_{4} (\omega ) - P_{2} (\omega )P_{3} (\omega )}} \\ \end{aligned}$$

where

5 $$\begin{aligned} P_{1} (\omega ) & = - \omega^{2} B_{O} ( - \omega^{2} ) \\ P_{2} (\omega ) & = B_{E} ( - \omega^{2} ) \\ P_{3} (\omega ) & = \omega B_{E} ( - \omega^{2} ) \\ P_{4} (\omega ) & = \omega B_{O} ( - \omega^{2} ) \\ P_{5} (\omega ) & = \omega^{2} A_{O} ( - \omega^{2} ) \\ P_{6} (\omega ) & = - \omega A_{E} ( - \omega^{2} ) \\ \end{aligned}$$

Simultaneous calculations of the Eq. (4) for a suitable range of *ω* and plotting the obtained values into the (*k*_*P*_, *k*_*I*_) plane determine the stability boundary locus. The obtained curve together with the line *k*_*I*_ = 0 split the (*k*_*P*_, *k*_*I*_) plane into the stable and unstable regions. The decision if the respective region represents stabilizing or unstabilizing area can be done simply using a test point within each region. Nonetheless, the appropriate frequency gridding could represent a potential problem. Thus, the Nyquist plot based technique from (Söylemez et al. 2003) can be used for the improvement of the method. In this improvement, the frequency *ω* can be separated into several intervals within which the stability or instability can not change. The borders of such intervals are defined by the real values of *ω* which fulfill the equation:

6 $$\text{Im} \left[ {G(s)} \right] = 0$$

The obtained intervals could be helpful for the proper frequency scaling.

## Computation of (nominal) stability regions for PID controllers

Now, the issue of (nominal) feedback stabilization will be elaborated again, but for the case of ideal PID controller given by:

7 $$C(s) = k_{P} + \frac{{k_{I} }}{s} + k_{D} s = \frac{{k_{P} s + k_{I} + k_{D} s^{2} }}{s}$$

The principal idea for obtaining the relevant stability regions is to fix one controller parameter to a certain value and calculate the stability boundary locus using two remaining parameters analogously to the procedure presented in the previous “Computation of (nominal) stability regions for PI controllers” section.

The expression for the stability boundary locus in the (*k*_*P*_, *k*_*I*_) plane for a fixed value of *k*_*D*_ leads to a bit modified equations for proportional and integral gains:

8 $$\begin{aligned} k_{P} (\omega ,k_{D} ) & = \frac{{P_{5} (\omega )P_{4} (\omega ) - P_{6} (\omega )P_{2} (\omega )}}{{P_{1} (\omega )P_{4} (\omega ) - P_{2} (\omega )P_{3} (\omega )}} \\ k_{I} (\omega ,k_{D} ) & = \frac{{P_{6} (\omega )P_{1} (\omega ) - P_{5} (\omega )P_{3} (\omega )}}{{P_{1} (\omega )P_{4} (\omega ) - P_{2} (\omega )P_{3} (\omega )}} \\ \end{aligned}$$

where

9 $$\begin{aligned} P_{1} (\omega ) & = - \omega^{2} B_{O} ( - \omega^{2} ) \\ P_{2} (\omega ) & = B_{E} ( - \omega^{2} ) \\ P_{3} (\omega ) & = \omega B_{E} ( - \omega^{2} ) \\ P_{4} (\omega ) & = \omega B_{O} ( - \omega^{2} ) \\ P_{5} (\omega ) & = \omega^{2} A_{O} ( - \omega^{2} ) + \omega^{2} B_{E} ( - \omega^{2} )k_{D} \\ P_{6} (\omega ) & = - \omega A_{E} ( - \omega^{2} ) + \omega^{3} B_{O} ( - \omega^{2} )k_{D} \\ \end{aligned}$$

Note that the last two terms in (9) depend on derivative constant *k*_*D*_. From the viewpoint of practical computation, *k*_*D*_ is considered to be chosen and the corresponding set of boundary parameters *k*_*P*_, *k*_*I*_ is consequently calculated while this process is repeated for several selected values of *k*_*D*_. Thus, the final stability regions are successively plotted through the “(*k*_*P*_, *k*_*I*_) sections” in the (*k*_*P*_, *k*_*I*_, *k*_*D*_) space.

Alternatively, the stability boundary locus in the (*k*_*P*_, *k*_*D*_) plane for a fixed value of *k*_*I*_ can be computed. This scenario would change the Eqs. (8) and (9) to, respectively:

10 $$\begin{aligned} k_{P} (\omega ,k_{I} ) & = \frac{{P_{5} (\omega )P_{4} (\omega ) - P_{6} (\omega )P_{2} (\omega )}}{{P_{1} (\omega )P_{4} (\omega ) - P_{2} (\omega )P_{3} (\omega )}} \\ k_{D} (\omega ,k_{I} ) & = \frac{{P_{6} (\omega )P_{1} (\omega ) - P_{5} (\omega )P_{3} (\omega )}}{{P_{1} (\omega )P_{4} (\omega ) - P_{2} (\omega )P_{3} (\omega )}} \\ \end{aligned}$$

where

11 $$\begin{aligned} P_{1} (\omega ) & = - \omega^{2} B_{O} ( - \omega^{2} ) \\ P_{2} (\omega ) & = - \omega^{2} B_{E} ( - \omega^{2} ) \\ P_{3} (\omega ) & = \omega B_{E} ( - \omega^{2} ) \\ P_{4} (\omega ) & = - \omega^{3} B_{O} ( - \omega^{2} ) \\ P_{5} (\omega ) & = \omega^{2} A_{O} ( - \omega^{2} ) - B_{E} ( - \omega^{2} )k_{I} \\ P_{6} (\omega ) & = - \omega A_{E} ( - \omega^{2} ) - \omega B_{O} ( - \omega^{2} )k_{I} \\ \end{aligned}$$

Obviously, the final stability regions are given by the “(*k*_*P*_, *k*_*D*_) sections” in the (*k*_*P*_, *k*_*I*_, *k*_*D*_) space.

Nonetheless, the third option of obtaining the stability boundary, which consists in fixing *k*_*P*_ and calculating the curves in (*k*_*I*_, *k*_*D*_) plane, is not so straightforward as the previous two alternatives, because for this case it holds true:

12 $$P_{1} (\omega )P_{4} (\omega ) - P_{2} (\omega )P_{3} (\omega ) = 0$$

However, the stability region in the (*k*_*I*_, *k*_*D*_) plane for a fixed *k*_*P*_ can be acquired using the stability region in the (*k*_*P*_, *k*_*I*_) plane and (*k*_*P*_, *k*_*D*_) plane together as it has been presented in (Tan et al. 2006). In accordance with a linear programming based approach from (Ho et al. 1997), the stability region in the (*k*_*I*_, *k*_*D*_) plane under fixed *k*_*P*_ is a convex polygon which can be sometimes advantageous for easier plotting.

## Robust stabilization using PI controllers

So far, the previous Sections were focused only on nominal stabilization of controlled plants with fixed parameters. The following parts will deal with robust stabilization. It means that the plant whose coefficients can vary within given intervals (interval system) is considered to represent a controlled object and the aim is to find all controllers which assure stabilization of all possible members of the interval system family. The works (Tan and Kaya 2003; Tan et al. 2006; Fang et al. 2009) have improved a feedback stabilization technique using PI controllers also for interval plants simply by using its combination with the sixteen plant theorem (Barmish et al. 1992; Barmish 1994; Ho et al. 1998). The sixteen plant theorem itself says that a first order controller robustly stabilizes an interval plant:

13 $$G(s,b,a) = \frac{B(s,b)}{A(s,a)} = \frac{{\sum\nolimits_{i = 0}^{m} {\left[ {b_{i}^{ - } ,b_{i}^{ + } } \right]s^{i} } }}{{s^{n} + \sum\nolimits_{i = 0}^{n - 1} {\left[ {a_{i}^{ - } ,a_{i}^{ + } } \right]s^{i} } }};\quad m < n$$

where *b*_*i*_^−^, *b*_*i*_^+^, *a*_*i*_^−^, *a*_*i*_^+^ represent lower and upper bounds for parameters of numerator and denominator, if and only if it stabilizes its 16 Kharitonov plants, defined as:

14 $$G_{i,j} (s) = \frac{{B_{i} (s)}}{{A_{j} (s)}}$$

where *i*, *j* ∊ {1, 2, 3, 4}; and *B*_1_(*s*) to *B*_4_(*s*) and *A*_1_(*s*) to *A*_4_(*s*) are the Kharitonov polynomials for the numerator and denominator of the interval plant (13).

Recall that the construction of Kharitonov polynomials e.g. for the numerator interval polynomial:

15 $$B(s,b) = \sum\limits_{i = 0}^{m} {\left[ {b_{i}^{ - } ;\;b_{i}^{ + } } \right]s^{i} }$$

is based on the use of the lower and upper bounds of interval parameters in compliance with the principle (Kharitonov 1978):

16 $$\begin{aligned} B_{1} (s) & = b_{0}^{ - } + b_{1}^{ - } s + b_{2}^{ + } s^{2} + b_{3}^{ + } s^{3} + \cdots \\ B_{2} (s) & = b_{0}^{ + } + b_{1}^{ + } s + b_{2}^{ - } s^{2} + b_{3}^{ - } s^{3} + \cdots \\ B_{3} (s) & = b_{0}^{ + } + b_{1}^{ - } s + b_{2}^{ - } s^{2} + b_{3}^{ + } s^{3} + \cdots \\ B_{4} (s) & = b_{0}^{ - } + b_{1}^{ + } s + b_{2}^{ + } s^{2} + b_{3}^{ - } s^{3} + \cdots \\ \end{aligned}$$

Consequently, the robust stabilization of an interval plant directly follows from the simultaneous stabilization of all 16 fixed Kharitonov plants. Hence, the final area of stability for original interval plant is given by the intersection of all 16 related partial areas obtained individually using the techniques from the “Computation of (nominal) stability regions for PI controllers” section.

## Robust stabilization using PID controllers

Unfortunately, the sixteen plant theorem is not applicable for robust stabilization of interval systems by PID controllers as it is not valid anymore (Pujara and Roy 2001). However, the suitable method based on the generalized Kharitonov theorem and linear programming techniques has been presented e.g. in (Ho et al. 1998, 2001). This paper adopts the idea of Kharitonov segments used in the generalized Kharitonov theorem (Chapellat and Bhattacharyya 1989) and similar thirty-two edge theorem (Barmish 1994; Chapellat and Bhattacharyya 1989) and combines it with the stability boundary locus technique (Tan and Kaya 2003; Tan et al. 2006).

Consider an interval plant:

17 $$G(s,b,a) = \frac{B(s,b)}{A(s,a)} = \frac{{\sum\nolimits_{i = 0}^{m} {\left[ {b_{i}^{ - } ,b_{i}^{ + } } \right]s^{i} } }}{{\sum\nolimits_{i = 0}^{n} {\left[ {a_{i}^{ - } ,a_{i}^{ + } } \right]s^{i} } }};\quad m < n$$

and a PID controller (7).

The family of interval systems (17) is stabilized by a fixed PID controller if and only if each of sixteen segment plants related to the interval family is stabilized by the same PID controller (Ho et al. 2001).

The mentioned 16 segment plants are defined as:

18 $$G_{i,j} (s,\lambda ) = \frac{{B_{Si} (s,\lambda )}}{{A_{j} (s)}}$$

where *i*, *j* ∊ {1, 2, 3, 4}; *A*_1_(*s*) to *A*_4_(*s*) are the Kharitonov polynomials for the denominator of the interval plant (17); and *B*_*S*1_(*s*, *λ*) to *B*_*S*4_(*s*, *λ*) are four Kharitonov segments (Chapellat and Bhattacharyya 1989; Ho et al. 2001; Barmish 1994) which can be written as:

19 $$\begin{aligned} B_{S1} (s,\lambda ) & = \left[ {B_{1} (s),B_{3} (s)} \right] = \left( {1 - \lambda } \right)B_{1} (s) + \lambda B_{3} (s) \\ B_{S2} (s,\lambda ) & = \left[ {B_{1} (s),B_{4} (s)} \right] = \left( {1 - \lambda } \right)B_{1} (s) + \lambda B_{4} (s) \\ B_{S3} (s,\lambda ) & = \left[ {B_{2} (s),B_{3} (s)} \right] = \left( {1 - \lambda } \right)B_{2} (s) + \lambda B_{3} (s) \\ B_{S4} (s,\lambda ) & = \left[ {B_{2} (s),B_{4} (s)} \right] = \left( {1 - \lambda } \right)B_{2} (s) + \lambda B_{4} (s) \\ \end{aligned}$$

where *λ* ∊ 〈0, 1〉 and *B*_1_(*s*) to *B*_4_(*s*) are the Kharitonov polynomials for the numerator of the interval plant (17).

The computation of robustly stabilizing PID controllers can be performed as follows: First, a certain value of controller parameter *k*_*D*_ is chosen and fixed (alternatively, also the parameter *k*_*I*_ or *k*_*P*_ can be fixed according to “Computation of (nominal) stability regions for PID controllers” section, but the fixed *k*_*D*_ is supposed here). Then, the stability boundary for one of segment plants (18) is calculated for several sampled values of *λ* ∊ 〈0, 1〉 using the Eqs. (8) and (9). The intersection of the obtained areas in (*k*_*P*_, *k*_*I*_) plane gives the stability boundary locus for this specific segment plant. The calculations are repeated for all the remaining segment plants and the robust stability region for the original interval plant and chosen value of *k*_*D*_ is determined by the intersection of areas for all 16 segment plants. From the practical viewpoint, the curves for all sampled *λ* ∊ 〈0, 1〉 and all 16 segment plants can be plotted in one figure and intersection can be found at a time. Anyway, the whole process should be repeated for the other selected values of *k*_*D*_ and the very final robust stability region can be visualized by the simultaneous plotting of the “(*k*_*P*_, *k*_*I*_) sections” into one graph in (*k*_*P*_, *k*_*I*_, *k*_*D*_) space.

## Illustrative example: Robust stabilization of oblique wing aircraft

This Section is intended to practically demonstrate the theoretical results from the previous parts by means of the illustrative example.

The controlled plant is supposed to be given by the uncertain mathematical model of an experimental oblique wing aircraft from (Barmish 1994; Dorf 1974):

20 $$G(s) = \frac{{\left[ {54,\;74} \right]s + \left[ {90,\;166} \right]}}{{s^{4} + \left[ {2.8,\;4.6} \right]s^{3} + \left[ {50.4,\;80.8} \right]s^{2} + \left[ {30.1,\;33.9} \right]s + \left[ { - 0.1,\;0.1} \right]}}$$

and the aim is to find all robustly stabilizing PI and PID controllers.

### PI controller

The first of the Kharitonov plants constructed according to (14) is:

21 $$G_{1,1} (s) = \frac{54s + 90}{{s^{4} + 4.6s^{3} + 80.8s^{2} + 30.1s - 0.1}}$$

Corresponding even and odd parts defined in the plant (3) are:

22 $$\begin{aligned} & B_{E} ( - \omega^{2} ) = 90 \\ & B_{O} ( - \omega^{2} ) = 54 \\ & A_{E} ( - \omega^{2} ) = \omega^{4} + 80.8( - \omega^{2} ) - 0.1 \\ & A_{O} ( - \omega^{2} ) = 4.6( - \omega^{2} ) + 30.1 \\ \end{aligned}$$

**Fig. 2:**
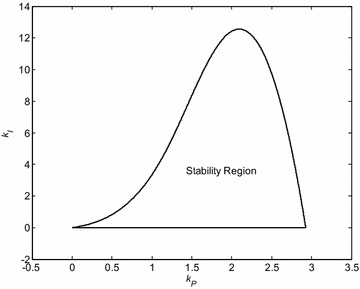
Stability region for PI controller and the first Kharitonov plant (21)

Simultaneous solving the Eqs. (4) and (5) for (22) and plotting the obtained results together with the line *k*_*I*_ = 0 into the (*k*_*P*_, *k*_*I*_) plane lead to the stability region which is visualized in Fig. 2. A decision on which part represents the area of stability can be simply done using an arbitrary pair (*k*_*P*_, *k*_*I*_) from this region, calculating the corresponding closed-loop characteristic polynomial and verifying its stability. In this case, the stabilizing area lies inside the depicted shape.

**Fig. 3:**
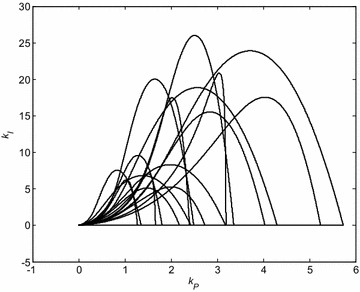
Stability regions for PI controller and 16 Kharitonov plants

The similar graphs can be plotted for remaining Kharitonov plants. The curves for all 16 Kharitonov plants are shown in Fig. 3.

**Fig. 4:**
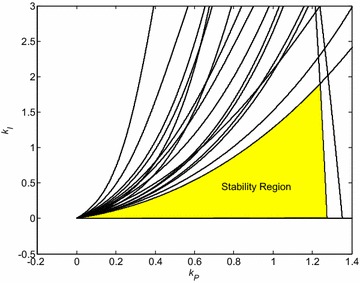
Robust stability region for PI controller and interval system (20)

The final robust stability region is determined by the intersection of regions for all 16 Kharitonov plants. It is zoomed and highlighted in Fig. 4.

Three PI controllers with specific position in relation to stability region from Fig. 4 have been chosen, namely:

23 $$C_{1} (s) = \frac{s + 1.5}{s}$$

24 $$C_{2} (s) = \frac{s + 1.277}{s}$$

25 $$C_{3} (s) = \frac{s + 0.5}{s}$$

**Fig. 5:**
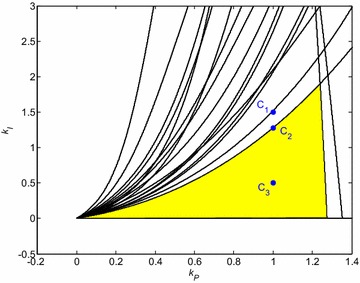
Robust stability region for PI controller and interval system (20) with highlighted positions of selected controllers

Their location is shown in Fig. 5.

**Fig. 6:**
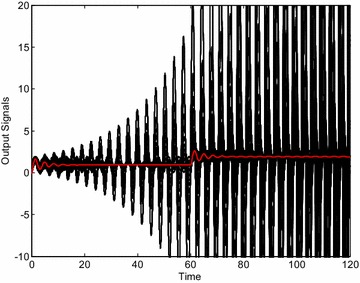
Output signals of “representative” plants for robustly non-stabilizing controller *C*_1_ (23)

The control results obtained by means of the selected controllers visually confirm the validity of robust stability region. The Fig. 6 shows the control responses of the loop with the controller *C*_1_ (23) and 729 “representative” systems from the interval family (20). Each interval parameter has been divided into 2 subintervals and thus these 3 values and 6 parameters have resulted in 3^6^ = 729 systems for simulation. Moreover, the red curve represents the output variable for the system with average values of uncertain parameters from (20):

26 $$G_{A} (s) = \frac{64s + 128}{{s^{4} + 3.7s^{3} + 65.6s^{2} + 32s}}$$

**Fig. 7:**
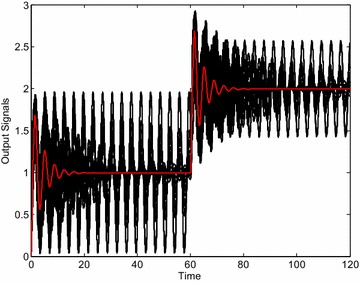
Output signals of “representative” plants for controller *C*_2_ (24) (stability border)

**Fig. 8:**
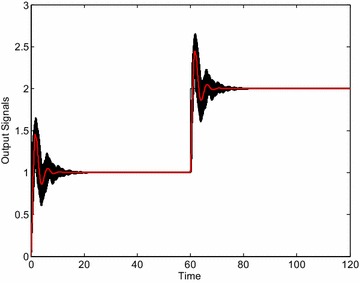
Output signals of “representative” plants for robustly stabilizing controller *C*_3_ (25)

As can be clearly seen, some members of the interval family (20) are not stabilized by the controller *C*_1_ (23) and thus the closed-loop system is really robustly unstable. The analogical simulations for controller *C*_2_ (24) and 729 + 1 “representative” systems lead to the set of control responses from Fig. 7. In this case, the closed-loop system is on the stability border for some member of the interval family (20) (the worst case) which concurs with the position of the controller *C*_2_ in Fig. 5. Finally, the same set of control responses is plotted in Fig. 8 for controller *C*_3_ (25). Now, the control loop is obviously robustly stable.

### PID controller

In this part, all robustly stabilizing PID controllers are going to be found for the same oblique wing aircraft model (20).

Initially, the derivative constant is chosen and fixed as *k*_*D*_ = 1. The first of the segment plants (18) is constructed using:

27 $$G_{1,1} (s,\lambda ) = \frac{{B_{S1} (s,\lambda )}}{{A_{1} (s)}} = \frac{{\left( {1 - \lambda } \right)B_{1} (s) + \lambda B_{3} (s)}}{{A_{1} (s)}}$$

where *B*_1_(*s*), *B*_3_(*s*) and *A*_1_(*s*) are relevant Kharitonov polynomials and *λ* ∊ 〈0, 1〉. More specifically:

28 $$G_{1,1} (s,\lambda ) = \frac{{B_{S1} (s,\lambda )}}{{A_{1} (s)}} = \frac{{\left( {1 - \lambda } \right)\left( {54s + 90} \right) + \lambda \left( {54s + 166} \right)}}{{s^{4} + 4.6s^{3} + 80.8s^{2} + 30.1s - 0.1}}$$

**Fig. 9:**
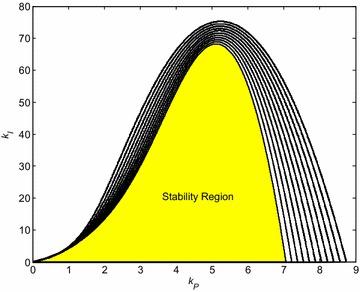
Stability region for PID controller (*k*_*D*_ = 1) and the first segment plant (28)

The stability boundary locus for the segment plant (28) is given by the intersection of the stability areas for several sampled values of *λ* ∊ 〈0, 1〉. The Fig. 9 shows 11 curves for the range *λ* = 0:0.1:1 and the highlighted area represents the intersection.

The same process can be analogously repeated for the remaining 15 segment plants and then the intersection of all 16 partial intersections would lead to the stability boundary locus for the original interval family (20) under the assumption of *k*_*D*_ = 1.

**Fig. 10:**
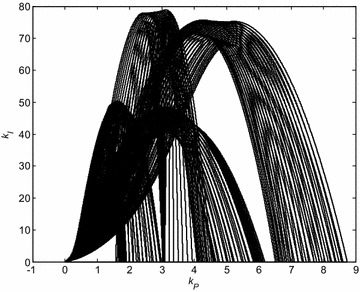
Stability regions for PID controller (*k*_*D*_ = 1) and all 16 segment plants

**Fig. 11:**
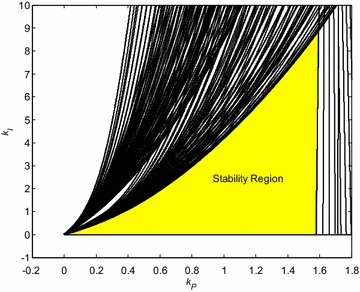
Robust stability region for PID controller with *k*_*D*_ = 1 and interval system (20)

The Fig. 10 presents the curves for all 16 segment plants and sampled *λ* = 0:0.1:1 in a single plot. The zoomed and highlighted intersection representing the robust stability region for closed loop containing PID controller with *k*_*D*_ = 1 and original interval system (20) is depicted in Fig. 11.

**Fig. 12:**
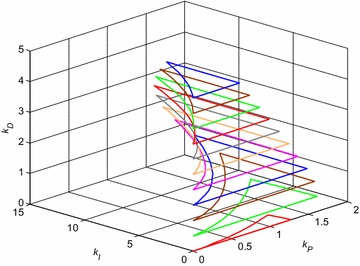
Final robust stability region for PID controller and interval system (20)

The whole previous process was repeated for the other values of derivative constant *k*_*D*_, more specifically for *k*_*D*_ = 0:0.5:5. The very final robust stability region visualized by means of corresponding eleven “(*k*_*P*_, *k*_*I*_) sections” in (*k*_*P*_, *k*_*I*_, *k*_*D*_) space is shown in Fig. 12.

The example of robustly stabilizing PID controller, which obviously lies inside the robust stability region from Fig. 12, can be chosen as:

29 $$C_{4} (s) = k_{P} + \frac{{k_{I} }}{s} + k_{D} s = 1 + \frac{0.5}{s} + 0.5s$$

**Fig. 13:**
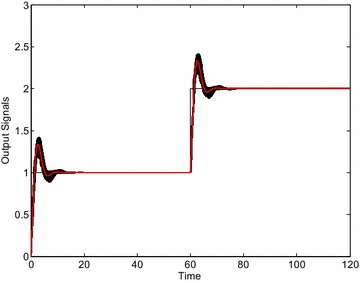
Output signals of “representative” plants for robustly stabilizing controller *C*_4_ (29)

The Fig. 13 demonstrates the set of control responses of the loop with the controller *C*_4_ (29) and 729 + 1 “representative” systems from the interval family (20) obtained analogously as in the previous PI control cases. As can be seen, the control loop is really robustly stable.

The intentional choice of a controller outside the robust stability region from Fig. 12 evidently leads to the robustly unstable closed control loop. The representative of such robustly non-stabilizing controller is e.g.:

30 $$C_{5} (s) = k_{P} + \frac{{k_{I} }}{s} + k_{D} s = 2 + \frac{0.5}{s} + 0.5s$$

**Fig. 14:**
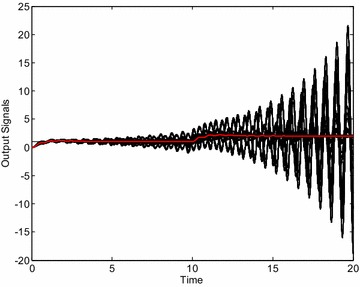
Output signals of “representative” plants for robustly non-stabilizing controller *C*_5_ (30)

Then, the corresponding set of control responses obtained under the same conditions as in the previous case is depicted in Fig. 14.

## Conclusion

The main aim of paper has been to present the improved method for computation of stabilizing controllers with the conventional structure on the basis of plotting the stability boundary locus in either P-I plane or P-I-D space. Now, thanks to the combination of the original method with stabilization of so-called segment plants, the modified technique can be conveniently used for determination of all possible robustly stabilizing PID controllers for interval plants. In the illustrative example, the model of an experimental oblique wing aircraft is considered as a controlled object. Two final robust stability regions have been computed and visualized, one for PI and the other for PID controller, and selected representatives from stable or even intentionally unstable areas have been chosen and used for supporting control simulations.
